# Inference of genetic ancestry from a multi-gene cancer panel in Colombian women with cancer

**DOI:** 10.1007/s10549-024-07557-7

**Published:** 2024-12-07

**Authors:** Yina T. Zambrano-O, Alejandro Mejía-Garcia, P. Daniela Morales, Hsuan Megan Tsao, Laura Rey-Vargas, Wendy Montero-Ovalle, Carlos A. Huertas-Caro, M. C. Sanabria-Salas, Julián Riaño-Moreno, Juliana L. Rodriguez, Carlos A. Orozco, Liliana Lopez-Kleine, I. King Jordan, Silvia J. Serrano-G

**Affiliations:** 1 Grupo de Investigación en Biología del Cáncer, Instituto Nacional de Cancerología, Cl. 1 #9-85, Bogotá, Colombia; 2 Universidad Nacional de Colombia – sede Bogotá, Bogotá, Colombia; 3 Department of Human Genetics, McGill University, Montreal, Canada; 4 Universidad Nacional de Colombia – sede Bogotá, Bogotá, Colombia; 5 Grupo de Investigación Clínica y Epidemiológica, Instituto Nacional de Cancerología, Bogotá, Colombia; 6 Departamento de Ginecología y Obstetricia, Universidad Nacional de Colombia – sede Bogotá, Bogotá, Colombia; 7 Grupo de Apoyo y Seguimiento Para La Investigación, Instituto Nacional de Cancerología, Bogotá, Colombia; 8 Departamento de Patología y Oncología Molecular, Instituto Nacional de Cancerología, Bogotá, Colombia; 9 Bioinformatics Department, Georgia Institute of Technology, Atlanta, USA; 10 Grupo de Investigación en Bioinformática y Biología de Sistemas, Departamento de Estadística, Universidad Nacional de Colombia, Bogotá, Colombia; 11 Division of Medical Oncology and Hematology, Department of Medicine, Princess Margaret Cancer Centre, University Health Network, University of Toronto, Toronto, ON M5G 2C1 Canada; 12 Faculty of Medicine, Universidad Cooperativa de Colombia, Villavicencio, Colombia

**Keywords:** Admixed populations, Diversity, Genetic ancestry, Clinical panels

## Abstract

**Introduction:**

Cancer health disparities among racial and ethnic populations significantly burden health systems due to unequal access to early detection, treatment, and healthcare resources. These disparities lead to worse outcomes and increased costs from delayed diagnoses, advanced treatments, and prolonged care. Genetic differences can also influence cancer susceptibility and treatment response, thus analyzing genetic ancestry is essential for uncovering genetic factors that may contribute to these disparities. Utilizing data from clinical multigene cancer panels to infer genetic ancestry offers a valuable approach to understand population structure and the impact of individual ancestries in development of complex diseases.

**Aim:**

To evaluate the accuracy of global ancestry inference using genetic markers from the TruSight™ Hereditary Cancer Panel, which was used to investigate hereditary cancer syndromes in a cohort of 116 female cancer patients at the Colombian National Cancer Institute. Additionally, to compare these results with genetic ancestry estimations from traditional genome-wide markers.

**Results:**

Our results demonstrate a strong correlation between global genetic ancestry inferred with markers captured from TruSight^TM^ panel (4785 markers) and Whole Genome Sequencing (WGS, 8 million markers in admixed populations. The correlation values were 0.96 (*p* < 0.0001) for the Native American and European ancestry components, and 0.99 (*p* < 0.0001) for the African ancestry fraction. Genetic ancestry mean proportions in the Colombian cohort were 45.7%, 46.2%, and 8.11% for the European, the Native American, and the African components, respectively.

**Conclusion:**

This study demonstrates the accuracy of ancestry inference from clinical panel data offering a promising approach for understanding cancer health disparities in admixed populations.

**Supplementary Information:**

The online version contains supplementary material available at 10.1007/s10549-024-07557-7.

## Introduction

Cancer incidence and mortality rates differ across population groups [1]. These disparities may be partially attributed to genetic factors, including variations in population genetic structure and frequencies of hereditary genetic predisposition [2, 3]. The integration of genetic ancestry in genomic research methods in cancer has improved genetic risk prediction models [4–6] and facilitated the exploration of the association between genetic ancestry and cancers [7]. For instance, European ancestry (EUR) has been associated with a higher breast cancer risk in the U.S., while Native American ancestry (NAM) is linked with protection against breast cancer in Colombian and Mexican women [8]. Ancestral background also influences gene expression in breast tumors, impacting cancer biology in admixed populations [9]. Despite these findings, many studies rely on self-reported race and ethnicity, which may not accurately represent an individual’s genetic background.

Technologies such as whole-exome sequencing (WES), RNA sequencing, and targeted panels have become indispensable tools for characterizing tumors, defining molecular profiles, and uncovering genetic variations associated with tumor initiation and progression. While primarily used in research, these tools are increasingly being integrated into clinical trials and institutional programs facilitating targeted therapies. These advancements have propelled the construction of public genomic databases, and the development of methods for inferring genetic ancestry from WES [10], RNA-sequencing data [11, 12], and tumor-targeted panels [12, 13]. Such methods have significantly improved our understanding of genetic ancestry and its influence on cancer. However, a critical limitation in this approach is the underrepresentation of Latin American patients on public genomic databases. Large cohort studies have included fewer than 2% of participants from Latin America [14], which limits the generalizability of findings to these populations.

Molecular characterization tools like WES and RNA-seq remain largely restricted to the research field and are not widely accessible in Latin America. Colombia faces significant limitations due to resource constraints, limiting the generation of genomic data for comprehensive studies, including ancestry estimation. However, germline genetic testing using multi-gene panels is becoming increasingly accessible in clinical settings, particularly for patients suspected of hereditary cancer. Leveraging these data to focus on specific genetic markers offers an opportunity to integrate genetic ancestry information into each patient’s clinical profile. This approach can potentially uncover novel associations between genetic ancestry and molecular-clinical features, which is particularly relevant for characterizing admixed populations. This approach allows for broader patient inclusion, enabling the study of a specific group with relevant genetic backgrounds and offering a practical alternative for advancing cancer research in these populations.

In this study, we demonstrated that genetic markers from a germline multi-gene cancer panel, routinely used to diagnose hereditary cancer syndromes at the Colombian National Cancer Institute (INC-Col), could accurately infer continental genetic ancestry proportions.

## Materials and methods

### Study samples

Patients diagnosed with triple negative breast cancer (TNBC) and high grade serous ovarian cancer (HGSOC) who were enrolled in the Hereditary Cancer Program at the Colombian National Cancer Institute (NCI-Col) between 2018 and 2023 were included in this study. As part of the program, each patient received genetic counseling and underwent germline testing following the National Comprehensive Cancer Network (NCCN) guidelines (2018–2023) [15, 16]. Germline testing was conducted using a standardized and validated next-generation sequencing (NGS) method. Specifically, we used the TruSight™ Hereditary Cancer Panel (Cancer Panel) which includes 105 genes (customized probe panel reference #20011891; Illumina Inc., San Diego, USA) (Supplemental Table S1). Testing was performed in our diagnostic laboratory using a MiSeq instrument (Illumina Inc., San Diego, CA) [17]. Details about germline DNA extraction, library preparation, and sequencing assays have been previously described [17]. All patients provided written informed consent, and both clinical data and biological samples were collected. Ethical approval for the study was obtained from the Ethics Committee of the NCI-Col, ensuring full compliance with ethical standards and patient privacy protections.

### Read mapping and variant calling

**Fig. 1:**
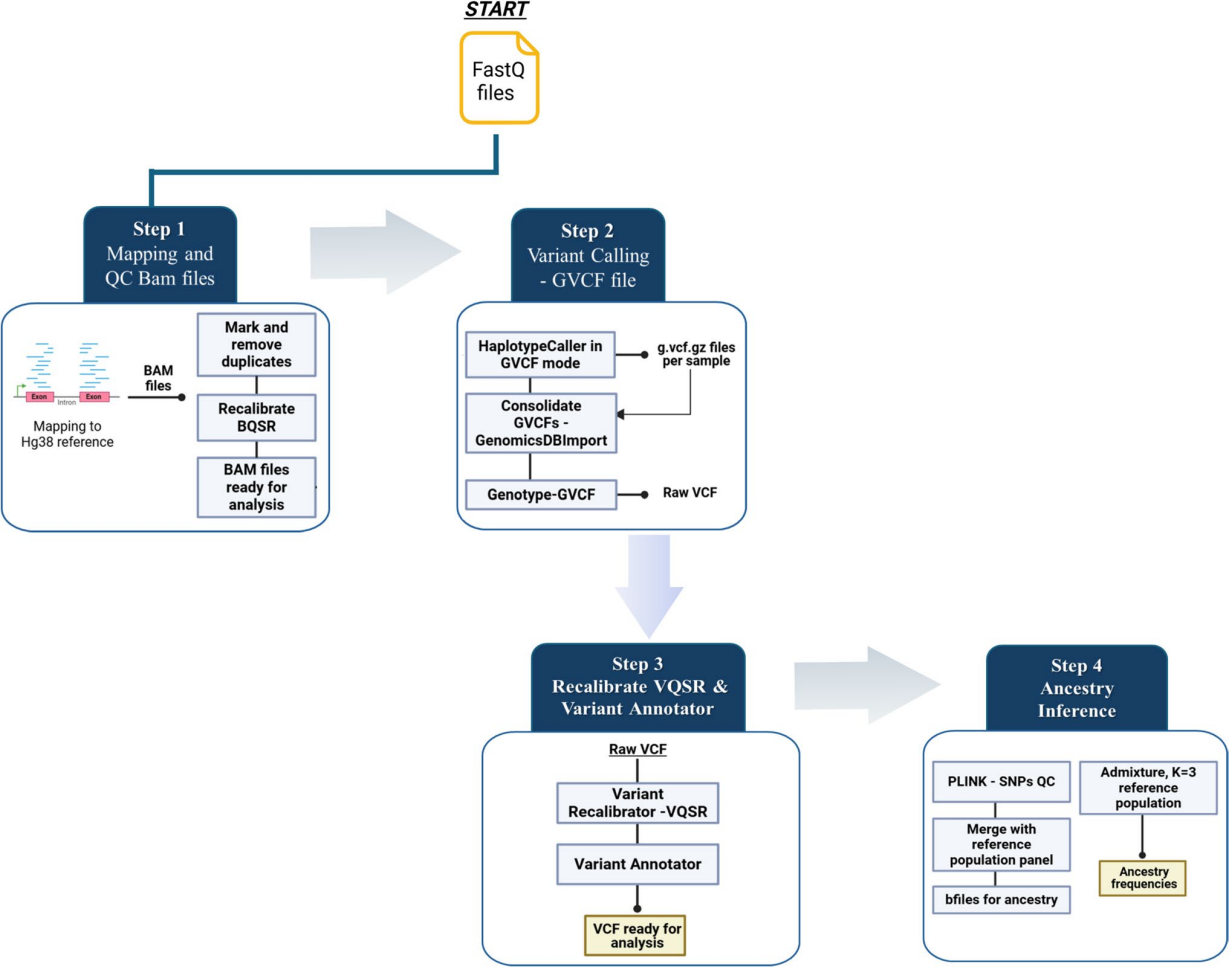
Pipeline employed in this study: Step 1: Minimap2 was used to map reads to the Hg38 human reference genome, and Picard Tools for marking duplicates in BAM files. BQSR was performed with GATK 4.2.2.0. Step 2: GATK v4.2.2.0 was used for variant calling in GVCF mode. Step 3: Variant quality score recalibration and variant annotation were performed using GATK v4.2.2.0. Step 4: PLINK and Admixture analysis were utilized for downstream analysis

Our analysis was based on the FASTQ files generated from the sequencing of the Cancer Panel previously described [17]. The bioinformatic pipeline is summarized in Fig. 1. Read mapping to Hg38 human reference genome was done with Minimap2 [18]. Picard Tools [19] was used for marking and removing duplicates. Following the workflow recommended in Best Practices of The Broad Institute’s Genome Analysis Toolkit (GATK) for variant discovery analysis in germline DNA, we applied base-quality score recalibration (BQSR), variant calling and filtering for SNPs/INDELS using the GATK platform (v4.2.2.0.) [20].

For variant quality score recalibration (VQSR), we used the following metrics: Quality by Depth (QD), Mapping Quality (MQ), Read Position Rank Sum (ReadPosRankSum), and the likelihood that a variant represents a true genetic variant. These metrics were applied to eliminate readings with low values and poorly mapped regions, and variants based on minimum coverage. Finally, we applied VQSR to calculate the variant quality score log-odds (VQSLOD) and filtered out bad quality variants, as specified by GATK best practices protocols.

### Quality control for markers in Colombian samples using cancer panel data

After applying filters to remove low-confidence variants from the sequencing data obtained with the Cancer Panel previously described, we retained 9958 SNPs in 117 Colombian samples. We performed the following quality control (QC) filters using PLINK 2.0 [21, 22]: Per-marker QC filters [minor allele frequency ≥ 2.5%, missingness ≤ 1.5%, linkage disequilibrium (LD) pairwise *r*^2^ ≤ 0.1, removal of ambiguous C/G and A/T SNPs, genotype calling rate (–geno 0.1), and Hardy–Weinberg equilibrium (–hwe 0.001)]. Per-individual QC filters [sample call rate of at least 75% for each individual (–mind 0.25)]. After applying these filters, one patient was removed, resulting in a final cohort of 116 female patients with 4785 SNPs remaining for analysis.

### Informativeness and distribution of SNPs captured from cancer panel data in Colombian samples

To evaluate the informativeness of the set of markers captured from the Cancer Panel, we estimated the allele frequency for 4785 SNPs per ancestral population obtained from the 1000 Genome Project (1KGP) and Human Genome Diversity Panel (HGDP) [23, 24]: Native American NAM), European (EUR), and African (AFR) populations (Table S2), and computed pairwise delta (absolute allele frequency difference) using the scikit-allele Python package. This analysis aimed to investigate which SNPs captured from the panel are the most informative. We identified variants with a delta > 0.5 for at least two of the three reference populations (Table S2. Their distribution across autosomal chromosomes was computed. Finally, we conducted a Principal Component Analysis (PCA) using PLINK 2.0 to evaluate how effectively the Cancer Panel differentiates individuals from different continental populations based on genetic variation.

### Genetic ancestry inference in the reference populations

To perform a supervised analysis, we retrieved VCF files from the 1000 Genome Project (1KGP) and Human Genome Diversity Panel (HGDP) [23, 24], for a total of 969 individuals from three different continental populations, EUR, AFR and NAM (Table S2). Binaries plink files from our cohort were merged with the 1KGP and HGDP samples using PLINK 2.0. A final set of 4785 SNPs cancer panel markers captured within the cohort of 116 Colombian patients was used to estimate genetic ancestry using Admixture 1.3 [25] in supervised mode with k = 3 populations (EUR, AFR, NAM).

### Genetic ancestry inference validation using WGS data

To evaluate the accuracy of the genetic ancestry inferred with the Cancer Panel, we compared these estimates with those inferred with WGS (gold standard). For this purpose, we pre-processed the reference population VCFs obtained from WGS (1KGP and HGDP, 24 M variants) using the same PLINK 2.0 QC parameters described above, resulting in a final VCF file with 8.4 M SNPs. We then estimated genetic ancestry using Admixture 1.3 in a supervised analysis with *k* = 3 ancestral reference populations (EUR, NAM, AFR).

Pearson correlations were computed between the genetic ancestry estimates inferred on individuals from reference populations (1 kg and HGDP samples, *n* = 969), using cancer panel markers (4,785 SNPs, restricted from WGS) and WGS (8 million SNPs). Additionally, we computed the Cosine similarity [13] between each ancestral component (EUR, NAM, and AFR) in admixed populations present in the reference panel (PUR, MXL, PEL, ACB, ASW) (Table S2). This analysis was performed to validate our panel's ability to capture diverse levels of admixture.

### Global ancestry proportions inferred from cancer panel in Colombian samples

Finally, global ancestry estimation was conducted in our cohort using the final VCF file containing 4785 SNPs to determine ancestry proportions with the Admixture 1.3 software. A supervised analysis was performed using three reference populations: European (EUR), Native American (NAM), and African (AFR), with *k* = 3 ancestral components.

## Results

### Markers’ informativeness and distribution

**Fig. 2:**
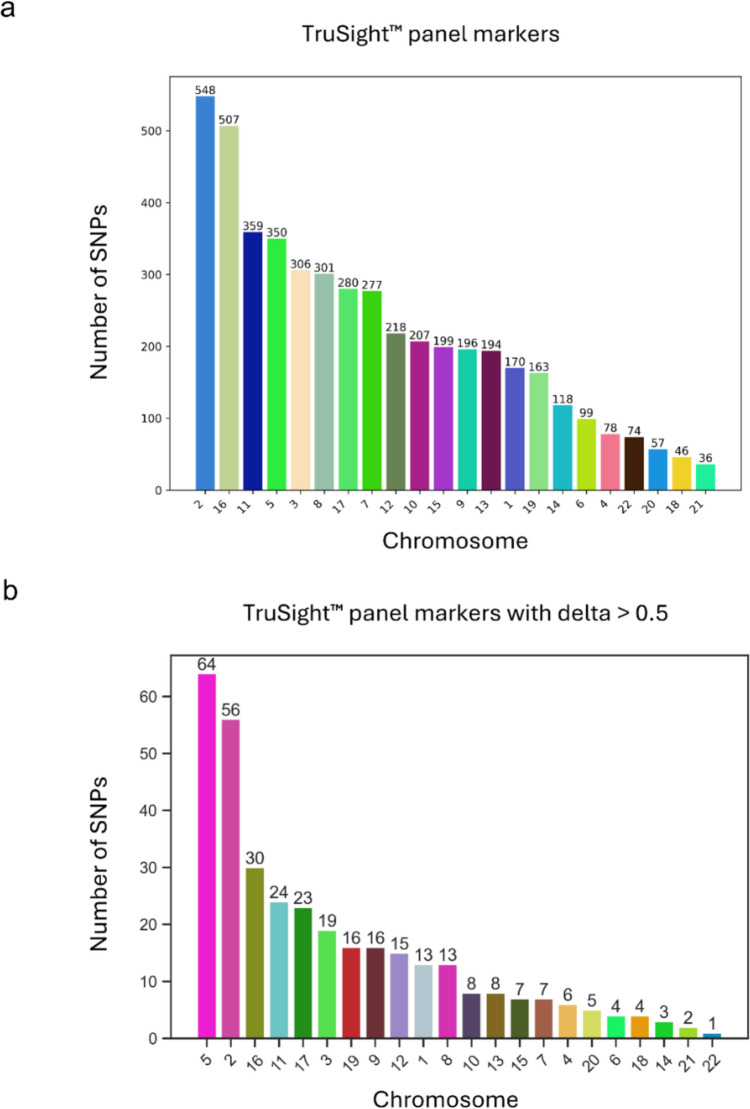
Distribution of the variants across the genome. **A** bar plot showing the distribution of variants across the genome captured by the TruSight^TM^ panel. **B** distribution of ancestry informative markers with delta > 0.5 in at least two out of three continental populations

Among 116 Colombian women in this study, we obtained 4785 SNPs with the Cancer Panel. We examined the distribution of these SNPs across the genome and observed their presence along the 22 autosomal chromosomes (Fig. 2a). Subsequently, to investigate the informativeness of the detected markers, we computed a pairwise delta between the ancestral populations (NAM, EUR and AFR. Table S2). We identified 344 variants with a delta > 0.5, representing the most informative SNPs among the 4785 selected from the Cancer Panel (Fig. 2b).

**Fig. 3:**
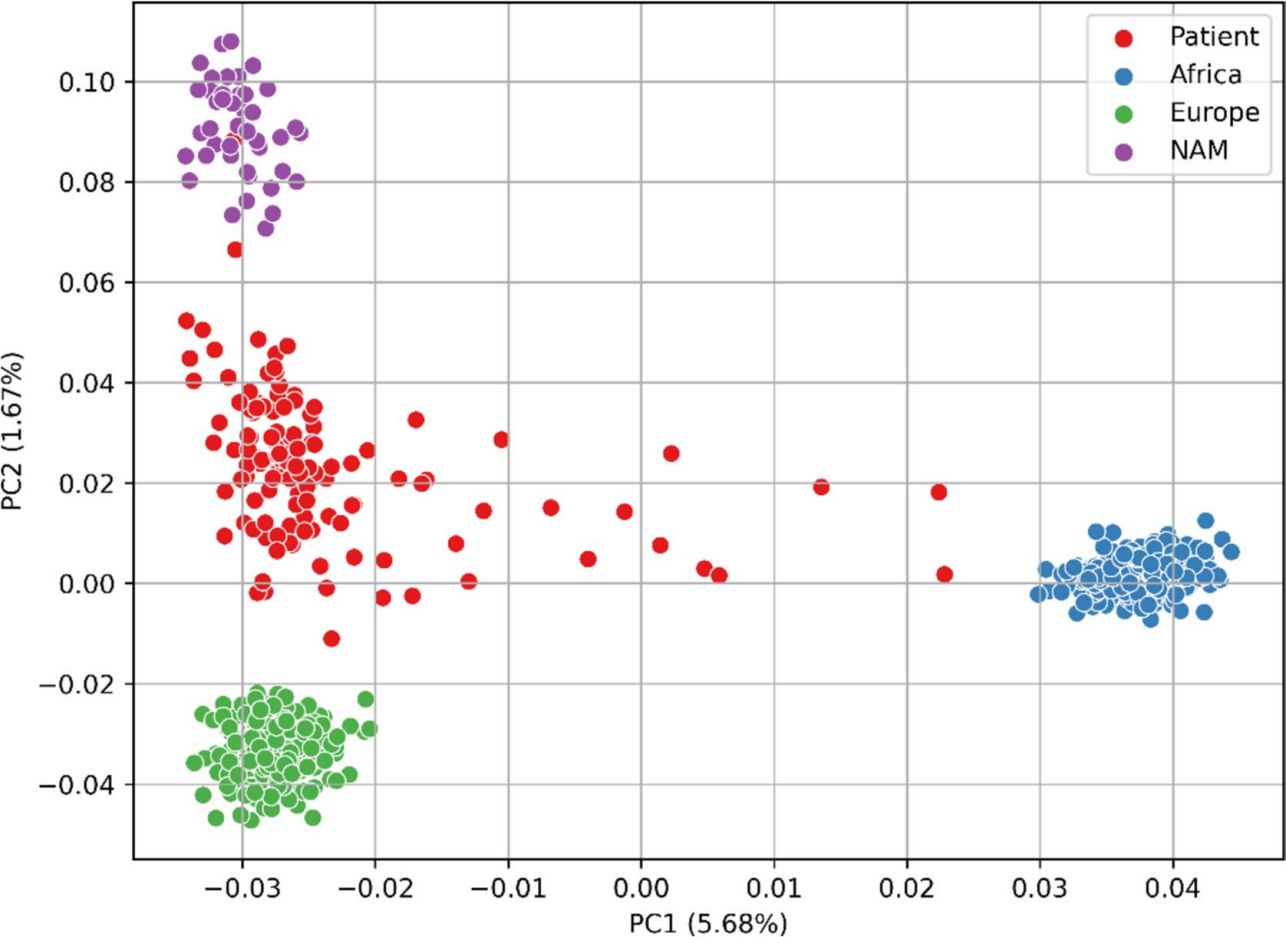
PCA with TruSight^TM^ panel markers. PCA was performed using 4785 variants from the Cancer Panel, which includes 116 patients from the cohort (represented as red dots). In addition to our cohort, 969 reference individuals from 1KGP and HGDP were included as reference populations. Patients are placed between the three ancestral populations, demonstrating the ability of these selected SNPs from Cancer Panel are effectively capture the genetic composition of the Colombian population by positioning the patients within the genetic continuum of the three ancestral populations: European (EUR), Native American (NAM), and African (AFR)

After confirming the genome-wide distribution and differentiation power of the Cancer Panel, we performed a principal component analysis (PCA) using a full set of 4785 SNPs. This analysis enabled us to compare the spatial distribution of the 116 patients from our cohort based on their genetic component, contrasting them with three continental reference populations: EUR, NAM, and AFR (Fig. 3).

### Genetic ancestry inference validation using WGS data

**Fig. 4:**
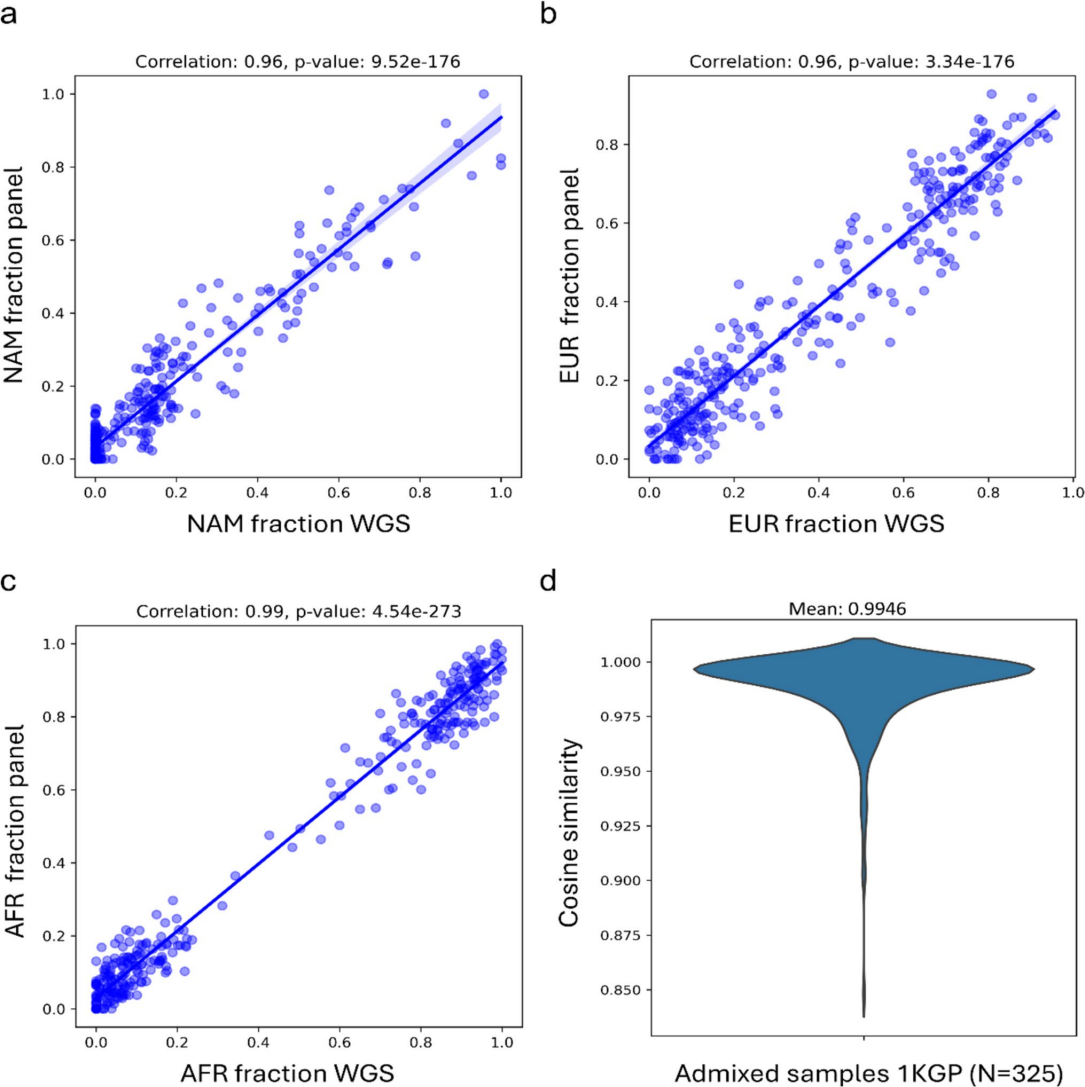
Correlation of genetic ancestry inference using the cancer panel markers (4785 markers) with ancestry inferred using WGS dataset (8 million markers). Statistically significant correlation of 0.96 for Native American (NAM) ancestry (panel **A**), 0.96 for European (EUR) ancestry (panel **B**), and 0.99 for African (AFR) ancestry (panel **C**) between WGS and cancer panel markers. Each blue dot in the graphs represents an admixed individual, while the blue straight lines indicate the fitted linear regression to the data points. Admixed samples in 1KGP showed a mean Cosine similarity of 0.99, demonstrating a good agreement between ancestry proportions inferred by the Cancer Panel and WGS

To validate the accuracy and representativeness of the full set of 4785 SNPs from the Cancer Panel for global ancestry estimation in different populations, we calculated the Pearson correlation between ancestry inferred using the cancer panel markers (4,785 markers) with ancestry inferred using the complete WGS dataset, which includes approximately 8 million SNPs, in admixed populations (PUR, ACB, MXL, and ASW). We found a statistically significant correlation of 0.96 (*p* < 0.0001) for both NAM (Fig. 4a) and EUR (Fig. 4b) ancestries, and 0.99 (*p* < 0.0001) for AFR ancestry (Fig. 4c). These results confirm that the markers identified by the Cancer Panel are accurate for global ancestry estimation in admixed individuals. Moreover, the calculated Cosine similarity estimates (Fig. 4d) showed a high concordance between ancestry proportions inferred by our panel compared to WGS (mean = 0.99).

### Global ancestry proportions inferred from cancer panel in Colombian samples

Finally, global ancestry estimation was performed using a VCF file containing 4785 SNPs to infer global ancestry proportions with admixture software using 3 ancestral populations as reference (Table 1). The mean for all patients in EUR, AFR, and NAM were 45.7% EUR, 8.11 AFR%, and 46.2 NAM%.

**Table 1:** Global Ancestry proportions in Colombian samples

Group	European ancestry (mean)	SD	African ancestry (mean)	SD	Native American ancestry (mean)	SD
All patients	0.457	0.131	0.0811	0.121	0.462	0.142

*SD* standard deviation

## Discussion

Incorporating genetic ancestry information in the clinical management of cancer patients has gained increasing importance in recent years. However, in many studies, genetic ancestry is commonly inferred from genotyping arrays, which are not standard clinical practice. With the increasing use of NGS (WES and RNA-seq) in clinical care, new bioinformatic protocols to infer genetic ancestry using these data have been implemented. To date, this is the first study of genetic ancestry in the Latino population using a clinical panel for inferring genetic ancestry. We demonstrated the reliability of variants within 105 cancer-predisposing genes for ancestry inference and population structure detection. Importantly, the ancestral fractions inferred with our panel closely align with those reported in previous studies of the Colombian population [26, 27], further supporting the panel’s accuracy.

Furthermore, our results revealed a strong correlation between global ancestry estimates derived from our Cancer Panel and those from WGS across admixed populations of the 1KGP dataset (Fig. 4). Previous studies using tumor profiling panels such as FoundationOne CDx, and the MSK-IMPACT have shown that approximately 5000 markers are sufficient for accurate genetic ancestry inference [12, 13]. However, these panels are sequenced from tumoral DNA, which can harbor alterations such as mutations, copy number variations (CNVs), and loss of heterozygosity (LOH), affecting genetic ancestry estimation [28]. Hence, a key strength of our study relies on using a germline cancer panel, which minimizes bias introduced by somatic events. Additionally, we treated ancestry as a continuous variable rather than categorizing individuals in broad continental groups, an approach particularly relevant for admixed populations such as Colombia. This contrasts with studies focusing primarily on race/ethnic categories, which do not reflect the genetic composition of participants.

Finally, it is important to highlight that the integration of genetic ancestry data into clinical cancer care requires careful consideration of the associated ethical, legal, and social implications [24]. This methodology not only advances our understanding of genetic ancestry in admixed populations but also provides valuable insights into the spectrum of germline variants in Colombians and their potential influence on cancer risk disparities in the country. As cancer genomics continues to evolve, determining genetic ancestry in cancer studies will enhance our understanding of the relationship between genetic factors and cancer biology in admixed populations. This will increasingly become an essential component of personalized oncology, enabling clinicians to provide more tailored and effective care for their patients.

## Conclusion

In conclusion, our findings indicate that genetic ancestry can be accurately inferred from clinical genetic data, marking an important step toward the integration of genetic ancestry into clinical research. This integration not only enhances our understanding of disease mechanisms but also aids in identifying genetic factors influencing cancer outcomes and the spectrum of germline variants in Colombian population. These insights provide a deeper understanding of their potential impact on cancer risk differences within the country. This approach contributes to the development of more personalized and effective therapeutic strategies, ultimately enhancing cancer care in admixed populations.

## Limitations

Despite limitations in sample size. We acknowledge that the number of SNPs in the panel and the distance between them do not allow haplotype analysis such as local ancestry and IBD estimation.

## Supplementary Information

Below is the link to the electronic supplementary material.

Supplementary file1 (DOCX 23 KB)

## Data Availability

No datasets were generated or analysed during the current study.
