# Supplementary material for: Inference of genetic ancestry from a multi-gene cancer panel in Colombian women with cancer
Source: Breast Cancer Res Treat. 2024 Dec 7;210(2):251–9. doi: 10.1007/s10549-024-07557-7 (PMC11930861; doi:10.1007/s10549-024-07557-7)
Supplement: Supplementary file 1 — Supplementary file1 (DOCX 23 KB) [file 10549_2024_7557_MOESM1_ESM.docx]

Supplementary Materials - Inference of Genetic Ancestry using data from a Multi-gene Cancer panel in Colombian Women with Cancer

**Table S1**. Genes included in the TruSight™ Hereditary Cancer Panel.

| **Gen** | **Chromosome** |  | **Gen** | **Chromosome** |  | **Gen** | **Chromosome** |
| --- | --- | --- | --- | --- | --- | --- | --- |
| *AIP* | 11 |  | *FANCB* | X |  | *PPM1D* | 17 |
| *ALK* | 2 |  | *FANCC* | 9 |  | *PRF1* | 10 |
| *APC* | 5 |  | *FANCD2* | 3 |  | *PRKAR1A* | 17 |
| *ATM* | 11 |  | *FANCE* | 6 |  | *PTCH1* | 9 |
| *BAP1* | 3 |  | *FANCF* | 11 |  | *PTEN* | 10 |
| *BARD1* | 2 |  | *FANCG* | 9 |  | *RAD50* | 5 |
| *BLM* | 15 |  | *FANCI* | 15 |  | *RAD51C* | 17 |
| *BMPR1A* | 10 |  | *FANCL* | 2 |  | *RAD51D* | 17 |
| *BRCA1* | 17 |  | *FANCM* | 14 |  | *RB1* | 13 |
| *BRCA2* | 13 |  | *FH* | 1 |  | *RECQL4* | 8 |
| *BRIP1* | 17 |  | *FLCN* | 17 |  | *RET* | 10 |
| *BUB1B* | 15 |  | *GATA2* | 3 |  | *RHBDF2* | 17 |
| *CASR* | 3 |  | *GNAS* | 20 |  | *RUNX1* | 21 |
| *CDC73* | 1 |  | *GPC3* | X |  | *SBDS* | 7 |
| *CDH1* | 16 |  | *HNF1A* | 12 |  | *SDHA* | 5 |
| *CDK4* | 12 |  | *HRAS* | 11 |  | *SDHAF2* | 11 |
| *CDKN1B* | 12 |  | *KIT* | 4 |  | *SDHB* | 1 |
| *CDKN1C* | 11 |  | *MAX* | 14 |  | *SDHC* | 1 |
| *CDKN2A* | 9 |  | *MEN1* | 11 |  | *SDHD* | 11 |
| *CEBPA* | 19 |  | *MET* | 7 |  | *SLX4* | 16 |
| *CEP57* | 11 |  | *MLH1* | 3 |  | *SMAD4* | 18 |
| *CHEK2* | 22 |  | *MRE11A* | 11 |  | *SMARCB1* | 22 |
| *CYLD* | 16 |  | *MSH2* | 2 |  | *STK11* | 19 |
| *DDB2* | 11 |  | *MSH6* | 2 |  | *SUFU* | 10 |
| *DICER1* | 14 |  | *MUTYH* | 1 |  | *TMEM127* | 2 |
| *DIS3L2* | 2 |  | *NBN* | 8 |  | *TP53* | 17 |
| *EGFR* | 7 |  | *NF1* | 17 |  | *TSC1* | 9 |
| *EPCAM* | 2 |  | *NF2* | 22 |  | *TSC2* | 16 |
| *ERCC2* | 19 |  | *NSD1* | 5 |  | *VHL* | 3 |
| *ERCC3* | 2 |  | *PALB2* | 16 |  | *WRN* | 8 |
| *ERCC4* | 16 |  | *PDE4D* | 5 |  | *WT1* | 11 |
| *ERCC5* | 13 |  | *PHOX2B* | 4 |  | *XPA* | 9 |
| *EXT1* | 8 |  | *PMS1* | 2 |  | *XPC* | 3 |
| *EXT2* | 11 |  | *PMS2* | 7 |  |  |  |
| *EZH2* | 7 |  | *POLD1* | 19 |  |  |  |
| *FANCA* | 16 |  | *POLE* | 12 |  |  |  |

**Table S2**. Populations used as reference in the analysis.

| **Ethnicity** | **Population** | **Abbreviation** | **No. of samples** |
| --- | --- | --- | --- |
| European | Iberian, Toscani, British | IBS; TSI; GBR | 273 |
| African | Yoruba in Ibadan, Esan in Nigeria, Gambian Mandinka | YRI; ESN; GWD | 312 |
| Native American | Peruvian, Pima in Mexico, Maya in Mexico, Karitiana in Brazil, Surui in Brazil | PEL; Pima; Maya; Karitiana; Surui | 59 |
| Puerto Rican | Admixed population, Puerto Rican in Puerto Rico | PUR | 104 |
| African Caribbean in Barbados | Admixed population, African Caribbean | ACB | 96 |
| Mexican Ancestry in Los Angeles, California | Admixed population, Mexican Ancestry | MXL | 64 |
| African Ancestry in Southwest US | Admixed population, African Ancestry SW | ASW | 61 |
